# Impaired Peripheral Vascular Function Following Ischemic Stroke in Mice: Potential Insights into Blood Pressure Variations in the Post-Stroke Patient

**DOI:** 10.3390/pathophysiology31030036

**Published:** 2024-09-05

**Authors:** Gokhan Yilmaz, Jonathan Steven Alexander

**Affiliations:** 1 Molecular Cellular and Biomedical Sciences, CUNY School of Medicine, New York, NY 10031, USA; 2 Molecular & Cellular Physiology, Louisiana State University Health Sciences Center, Shreveport, LA 71103, USA; jonathan.alexander@lsuhs.edu

**Keywords:** ischemic stroke, blood pressure, hypertension, hypotension, vasodilation, vasoconstriction, peripheral vessels, myography, alpha-adrenergic receptors, middle cerebral artery occlusion

## Abstract

High systolic blood pressure and increased blood pressure variability after the onset of ischemic stroke are associated with poor clinical outcomes. One of the key determinants of blood pressure is arteriolar size, determined by vascular smooth muscle tone and vasodilatory and vasoconstrictor substances that are released by the endothelium. The aim of this study is to outline alterations in vasomotor function in isolated peripheral arteries following ischemic stroke. The reactivity of thoracic aortic segments from male C57BL/6 mice to dilators and constrictors was quantified using wire myography. Acetylcholine-induced endothelium-dependent vasodilation was impaired after ischemic stroke (LogIC50 Sham = −7.499, LogIC50 Stroke = −7.350, *p* = 0.0132, n = 19, 31 respectively). The vasodilatory responses to SNP were identical in the isolated aortas in the sham and stroke groups. Phenylephrine-induced vasoconstriction was impaired in the aortas isolated from the stroke animals in comparison to their sham treatment counterparts (Sham LogEC50= −6.652 vs. Stroke LogEC50 = −6.475, *p* < 0.001). Our study demonstrates that 24 h post-ischemic stroke, peripheral vascular responses are impaired in remote arteries. The aortas from the stroke animals exhibited reduced vasoconstrictor and endothelium-dependent vasodilator responses, while the endothelium-independent vasodilatory responses were preserved. Since both the vasodilatory and vasoconstrictor responses of peripheral arteries are impaired following ischemic stroke, our findings might explain increased blood pressure variability following ischemic stroke.

## 1. Introduction

Ischemic stroke is a devastating disease and the fifth leading cause of death in the United States. It is also one of the main causes of long-term disability, with an estimated 7.6 million annual stroke survivors. Stroke-related disability places a great burden on society [1]. For example, the estimated cost of stroke treatment and related morbidity cost in 2018 was USD 52.8 billion [2]. Despite the high impact of ischemic stroke on general public health, current therapies are limited to the restoration of cerebral blood flow by pharmacological thrombolysis or thrombectomy [3], and the prevention and prompt treatment of secondary insults. Despite numerous pharmacological therapies tested to prevent the sequelae of ischemic stroke, the experimental therapies have not been successfully translated to clinical settings [4]. While many therapeutic approaches target the neuronal death resulting from ischemia, a critical gap exists in the literature regarding the co-existing morbidities and their underlying mechanisms in stroke patients. These co-morbidities include dysregulation of blood pressure, cardiac dysfunction, and metabolic and immune alterations that are related to stroke [5,6]. These co-morbidities responsible for dysregulation of blood pressure triggered by cerebral ischemia and reperfusion are significant and require the development of novel treatment strategies [5,7,8].

It has been established that high systolic blood pressure [9] and increased blood pressure variability after the onset of ischemic stroke are associated with poor outcomes [7,10,11] and increased mortality in patients [12]. Although the definitive mechanisms are not established, blood pressure variability after ischemic stroke in patients might be associated with autonomic nervous dysfunction [13], cardiopulmonary (Bezold–Jarisch) reflex activation due to the stimulation of chemoreceptors in atria and pulmonary vessels, stroke-induced inflammation [14], alterations in body temperature [15], and pain related to the procedures and post-stroke pain syndrome. Systemic mean arterial blood pressure is determined by cardiac output and systemic vascular resistance. One of the key determinants of systemic vascular resistance is arteriolar size. Arteriolar size is further determined by the contractility of the vascular smooth muscle and the vasodilatory and vasoconstrictor substances that are released by the endothelial cells. Any change in the responses of either endothelial cells and/or vascular smooth muscle would alter the mean arterial blood pressure. Although clinical studies suggest that endothelium-dependent and independent vasodilation are impaired in stroke patients [16,17], the mechanisms that underlie impaired peripheral vascular responses in ischemic stroke remain unclear. Local alterations in the peripheral vascular responses such as endothelium-dependent and independent vasodilation and vascular smooth muscle contraction following ischemic stroke might be the underlying causes of the mean arterial blood pressure variability following ischemic stroke. Since blood pressure variability is a risk factor following ischemic stroke, it is of paramount importance to address the underlying mechanisms of impaired vascular responses in ischemic stroke.

The main underlying concept of the current study is that ischemic stroke is accompanied by one or more features of vascular dysfunction at remote sites of the body other than the brain itself. This contention is supported by three major lines of evidence. (a) It has been established that high systolic blood pressure [9] and increased blood pressure variability after the onset of ischemic stroke are associated with poor outcomes [7,10] and increased mortality in patients [12]. (b) Following ischemic stroke, patients exhibit impaired peripheral vasodilation due to endothelial dysfunction compared to hypertension-matched controls and healthy control groups [16]. (c) There is a correlation between the expression of proinflammatory cytokines and endothelial dysfunction observed in patients following ischemic stroke [18]. 

Although clinical studies document an association between ischemic stroke and endothelial-dependent vascular dysfunction at peripheral circulation, there are limited studies that address the mechanism behind vascular dysfunction following ischemic stroke. Additionally, there is a lack of established studies that identify and measure both vasoconstriction and endothelium-dependent as well as endothelium-independent vasodilation following an ischemic stroke. Consequently, the aim of this study is to outline the changes in vasomotor function in isolated peripheral arteries following ischemic stroke.

## 2. Materials and Methods

### 2.1. Animals 

All experimental procedures were performed on 6–14-week-old male wildtype C57Bl/6 mice. The animal experiments were performed according to the criteria outlined by the National Institutes of Health and were approved by the Institutional Review Board (or Ethics Committee) of the City College, City University of New York, IACUC (Protocol No.: 1121.A1 and 23 March 2023). A sham group exposed to a sham operation (n = 31) and an ischemic stroke group subjected to 1 h of middle cerebral artery occlusion (MCAO) followed by 23 h of reperfusion (n = 19) were used.

### 2.2. Ischemic Stroke Model 

The mice were anesthetized by inhaled isoflurane (Covetrus, Dublin, OH, USA). Transient (60 min) focal cerebral ischemia and reperfusion were induced by occlusion and reperfusion of the left middle cerebral artery (MCAo/R). A blunted tip of a 6–0 nylon monofilament was advanced to the level of the carotid bifurcation via the internal carotid artery until light resistance was felt. The monofilament was removed after 60 min of occlusion. In the sham group, these arteries were visualized but not disturbed. Following the stroke or sham surgery, lidocaine cream (Covetrus, Dublin, OH, USA) for local control of pain and Neosporin cream (Johnson and Johnson, Skillman, NJ, USA) for the control of wound infection was applied. Postoperative pain was alleviated by analgesic meloxicam (Covetrus, Dublin, OH, USA) 5 mg/kg sc. The body temperature of the mice was kept constant by a probe coupled with a thermo-blanket (ThermoStar, RWD, Shenzhen, China). At the end of the experiments, the mice were euthanized, and the brains were removed and then stained with 2% 2, 3, 5-triphenyl tetrazolium chloride (TTC, T8877, Sigma Aldrich, St. Louis, MO, USA) to confirm the production of an infarct [19]. NIH Image J was used for image processing and quantification of the percentage of the infarct area. 

### 2.3. Wire Myography 

The reactivity of the mouse thoracic aortic segments to different dilators and constrictors were quantified using wire myography (DMT, Hinnerup, Denmark) [20]. To achieve this, 2 mm long segments of the aorta were isolated 24 h after the onset of either procedure and were mounted in a small vessel wire myograph. The vessel rings were maintained in 5 mL organ baths with oxygenated physiological saline solution (95% O_2_ and 5% CO_2_) at 37.1 °C. The aortic rings were rinsed with a 60 mM KCl (P3911, Sigma Aldrich, St. Louis, MO, USA) solution for vascular smooth muscle activation and to determine the maximal contractile response. The aortic rings were then precontracted with 10^−6^ M phenylephrine (PE) (P6126, Sigma Aldrich, St. Louis, MO, USA) to obtain submaximal contraction, and after obtaining a stable plateau phase of contraction, the endothelium-dependent responses of the endothelium wire were assessed by increasing the cumulative concentrations of acetylcholine (ACh: 10^−9^–10^−4^ M)( A6625, Sigma Aldrich, St. Louis, MO, USA). Endothelium-dependent dilations were expressed as the percentage dilation from the precontraction response to 10^−6^ M PE. Endothelium-independent vasorelaxation was determined by the application of increasing cumulative concentrations of sodium nitroprusside (SNP; 10^−9^–10^−6^ M) (431451, Sigma Aldrich, St. Louis, MO, USA), while vascular smooth muscle contraction was determined by increasing cumulative concentrations of PE (10^−9^–10^−4^ M). PE, Ach, and SNP were selected to assess vascular reactivity based on the guidelines to measure vascular function [21]. ACh has been used to induce endothelium-dependent vasorelaxation and SNP is used as a classical nitric oxide donor [22]. PE is a selective alpha-1 receptor agonist that has been utilized for vascular smooth muscle contraction experiments [23].

### 2.4. Statistical Analysis

All values are reported as means ± SE. Comparisons between the two groups of animals or treatments were made by *t*-test, and comparisons among groups of more than 2 were analyzed by one-way ANOVA. Statistical analysis, nonlinear analysis of the dose response curves, curve fitting, and half maximal effective concentration (EC50) and half-maximal inhibitory concentration (IC50) values were performed using GraphPad Prism (version 10.2.3 (347), for MacOS, GraphPad Software, Boston, MA, USA, www.graphpad.com). A null hypothesis of EC50 and IC50 for the same datasets is tested. At *p* < 0.05, the null hypothesis is rejected and is reported as significant.

## 3. Results

### 3.1. Endothelium-Dependent Vasodilation Is Impaired Following Ischemic Stroke

**Figure 1:**
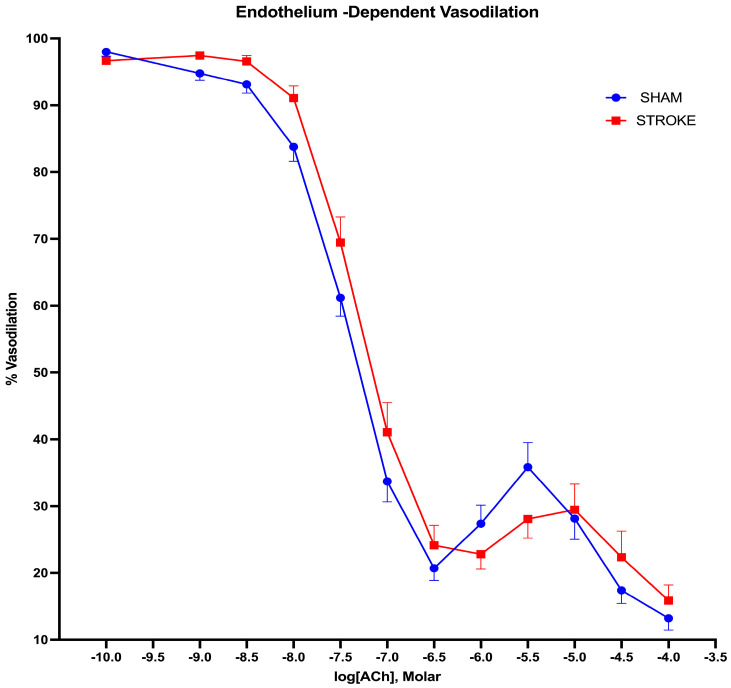
Endothelium-dependent vasodilation. 1 h brain ischemia followed by 23 h reperfusion resulted in impaired vasodilation in isolated mice thoracic aorta in response to increasing concentrations of acetylcholine (ACh) in organ bath (*p * = 0.0132, IC50 of ACh sham vs. stroke, n = 31, 19, respectively).

**Figure 2:**
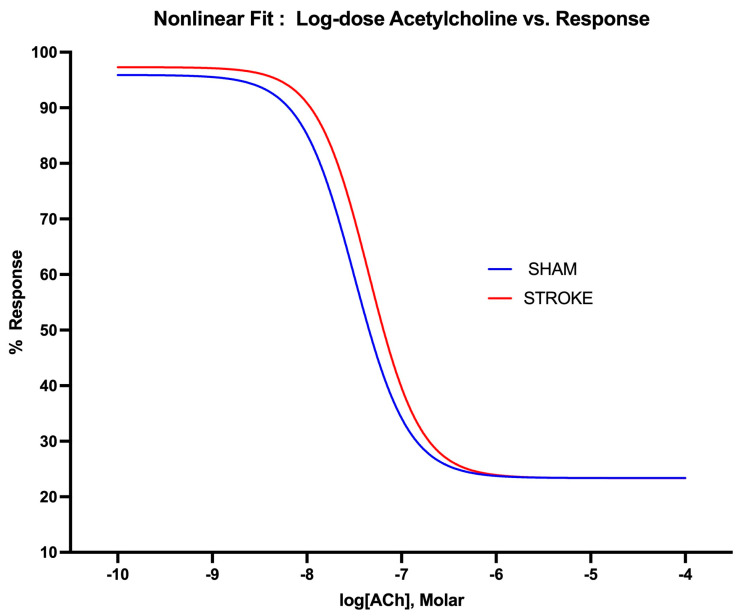
Nonlinear fitting of acetylcholine dose-response curve. IC50 is different for sham and stroke groups (*p * = 0.0132, IC50 of ACh sham vs. stroke groups, whereas top, bottom, and Hill Slope (*p* = 0.8569) are shared with the curves and are not significantly different for the sham and stroke group curves.

Endothelium-dependent nitric oxide-mediated vasodilation was assessed by increasing concentrations of acetylcholine. Acetylcholine-induced endothelium-dependent vasodilation was impaired after ischemic stroke in the thoracic aorta (Figure 1). The greatest difference in acetylcholine-induced vasodilatation was observed in the concentration range of 10^−8^ M to 10^−6^ M of acetylcholine between the stroke and sham groups. A 1.42-fold increase in the concentration of acetylcholine was required to achieve 50% of the vasodilation in the aortas isolated from the stroke mice in comparison to the sham treatment mice. The LogIC50s for ACh were different in the sham group vs. stroke group vessels (LogIC50 Sham = −7.499 vs. LogIC50 Stroke = −7.350, *p * = 0.0132, n = 19, 31, respectively) (Figure 2).

### 3.2. Endothelium-Independent Vasodilation Is Preserved Following Ischemic Stroke

**Figure 3:**
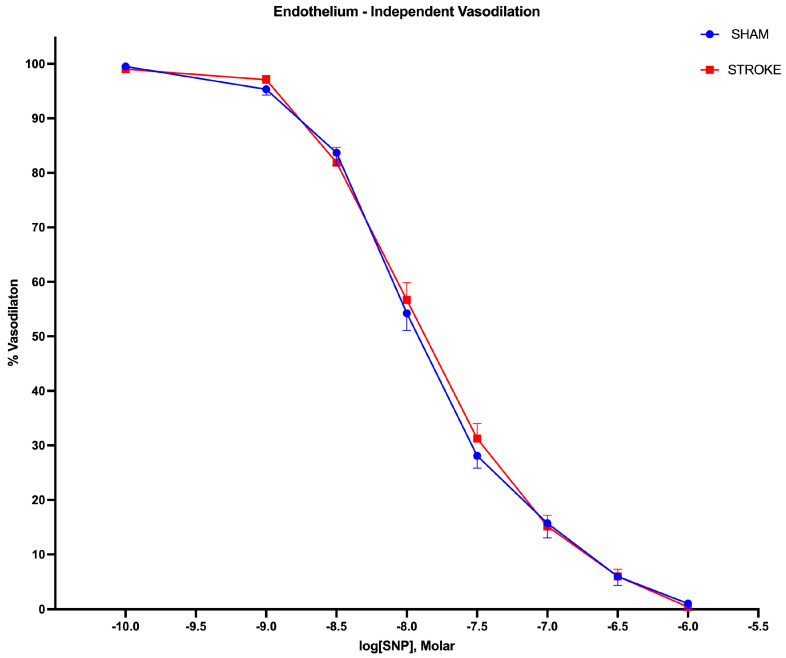
Endothelium-independent vasodilation. There were no differences in endothelium-independent vasodilation responses in aortas isolated from sham and stroke animals. 1 h brain ischemia followed by 23 h reperfusion did not change endothelium-independent vasodilation in isolated mice thoracic aorta in response to increasing concentrations of sodium nitroprusside (SNP), a nitric oxide donor, in organ baths. (*p* = 0.3637, IC50 of SNP sham vs. stroke, n = 31, 19, respectively).

**Figure 4:**
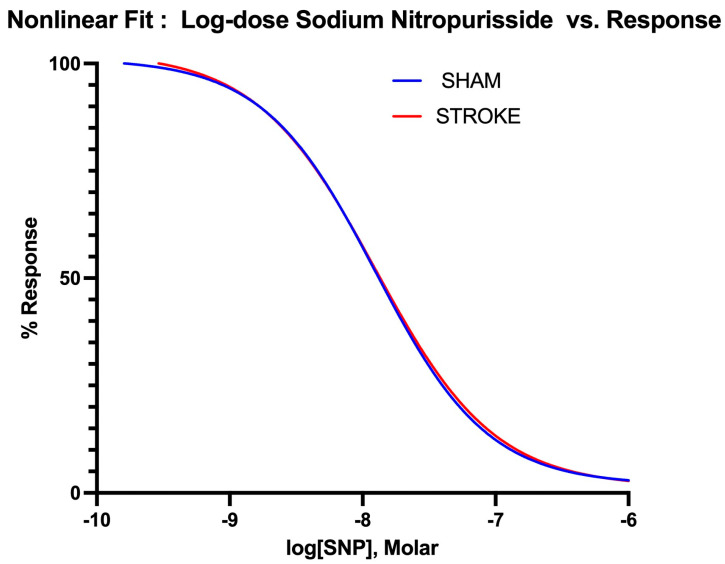
Nonlinear fitting of sodium nitroprusside dose-response curve. IC50, top, bottom and Hill Slope values are not different for sham and stroke groups *p* > 0.05.

Endothelium-independent vasodilation was assessed by the administration of increasing SNP, a non-enzymatic nitric oxide donor. The vasodilatory responses to the SNP were identical in isolated aortas in the sham and stroke groups, as shown in Figure 3 (Log IC50 Sham = −7.924 vs. Log IC50 Stroke = −7.875, *p* = 0.3637). There were no differences between the curves (Figure 4).

### 3.3. Vascular Smooth Muscle Contraction Is Impaired Following Ischemic Stroke

**Figure 5:**
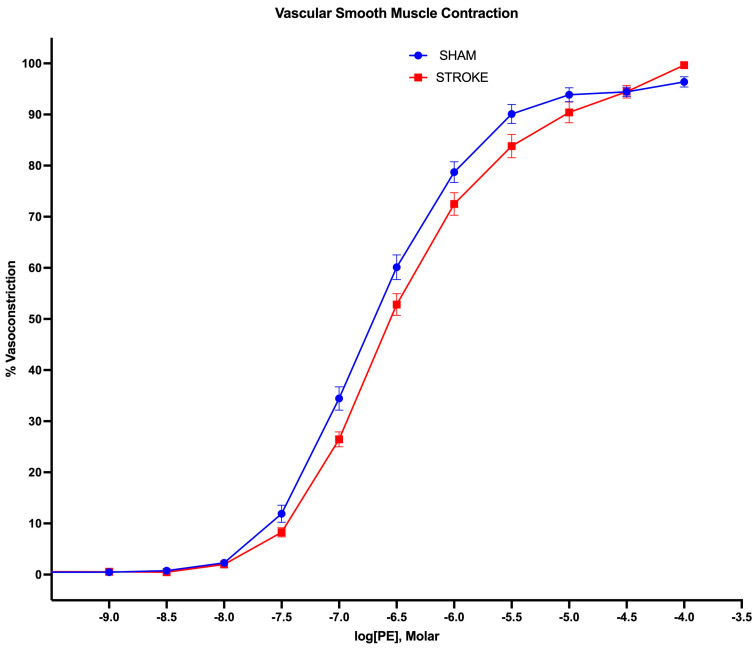
Vascular smooth muscle contraction. 1 h brain ischemia followed by 23 h reperfusion resulted in impaired vasoconstriction in isolated mice thoracic aorta in response to increasing concentrations of phenylephrine (PE) in organ bath (*p* < 0.0001, EC50 of PE sham vs. stroke, n = 31, 19, respectively).

**Figure 6:**
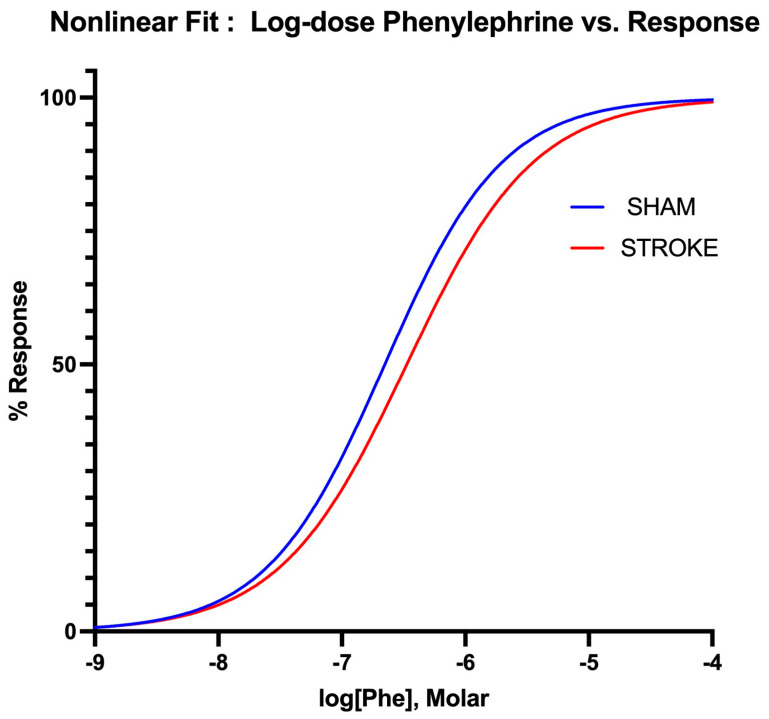
Nonlinear fitting of phenylephrine dose-response curve. EC50s is different for sham and stroke groups (*Sham* LogEC50 = −6.652 vs. *Stroke* LogEC50 = −6.475, *p* < 0.05), whereas top, bottom and Hill Slope are shared and not significantly different for the sham and stroke PE curves *p* > 0.05.

Vascular smooth muscle contraction in the aorta was assessed by the administration of increasing concentrations of alpha−1 receptor agonist phenylephrine. Phenylephrine-induced vasoconstriction was impaired in the aortas isolated from the stroke animals in comparison to their sham counterparts (Figure 5). The sham and stroke group dose response curves have significantly different LogEC50s (Sham LogEC50= −6.652 vs. Stroke LogEC50 = −6.475, *p* < 0.001) (Figure 6). This impairment was prominent in the concentration range of 10^−7^ M–10^−5^ M of phenylephrine concentration. A 1.5-fold increase in phenylephrine concentration was required to achieve 50% of maximum vasoconstriction in the aortas isolated from stroke animals in comparison to their sham counterparts (Figure 5).

### 3.4. Infarct Size

**Figure 7:**
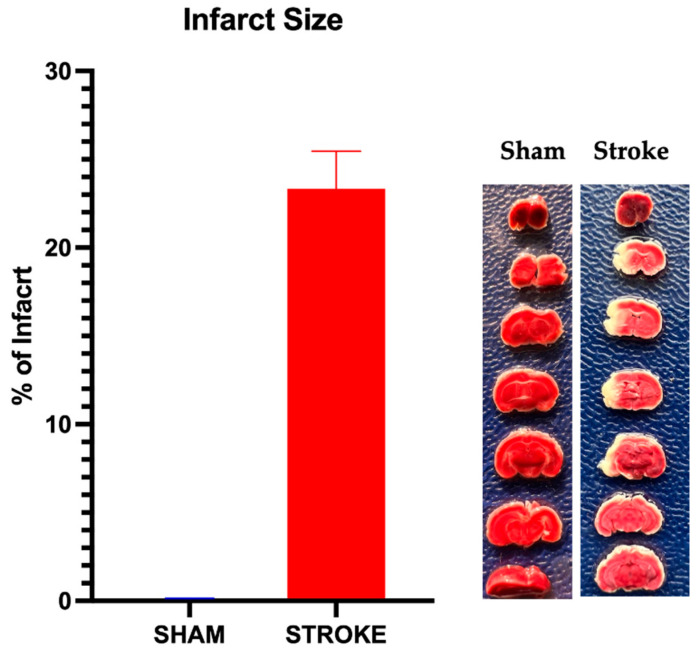
Infarct percentage after 1 h of middle cerebral artery occlusion and 23 h of reperfusion. Infarct percentage was 23.2 ± 1.5% (n = 19) assessed by TTC staining of brain sections. No infarct detected in sham group. Representative photographs of TTC staining of sham and stroke brains are shown.

Infarct size was calculated as the percentage of the largest cross-sectional area of infarct compared to the whole brain area for each brain sample. In the sham group, no infarct was observed. In the stroke group, 24 h after the induction of MCAO/R, an average infarct size of 23.2 ± 1.5% (n = 19) was observed. A representative of infarct vs. sham group brain samples with the mean infarct percentage of the stroke group is shown in Figure 7.

## 4. Discussion

Our study demonstrates that 24 h following ischemic stroke, peripheral vascular responses are impaired in remote arteries (aortas). The aortas isolated from the stroke animals exhibited both reduced vasoconstrictor and reduced endothelium-dependent vasodilator responses, while endothelium-independent vasodilatory responses were preserved. Our findings indicate the local dysregulation of peripheral vascular responses following ischemic stroke.

Endothelium-dependent vasodilator responses depend on a healthy endothelial cell layer and healthy nitric oxide production in endothelial cells. Our results indicate an impaired vasodilatory response to acetylcholine following ischemic stroke. Acetylcholine binds to its muscarinic receptor and activates eNOS, which in turn produces nitric oxide. Nitric oxide then diffuses to the underlying smooth muscle, activating guanylyl cylase, which leads to vasodilation [24]. An impairment of endothelium-dependent vasodilation in the presence of preserved endothelium-independent vasodilation indicates endothelial dysfunction following ischemic stroke. It is possible that direct endothelial cell damage, a decreased expression or activity of eNOS, low substrate availability, endogenous inhibition of eNOS, or reduced bioavailability of NO may contribute to the diminished vasodilatory responses observed following ischemic stroke [24].

Vascular smooth muscle tone is locally mediated by nitric oxide production in the endothelial cells. The impairment of endothelium-derived nitric oxide-dependent vasodilation leads to increased vascular resistance and increased blood pressure [25,26]. Our results might suggest a link between the ischemic stroke-induced endothelial damage and increased blood pressure observed in ischemic stroke [27]. Although impaired vasodilatory response in stroke patients was observed [28], whether this response is linked to the increased blood pressure increase associated with ischemic stroke has not been well defined. While increased blood pressure following ischemic stroke has been reported as a poor prognostic factor [29,30] and associated with all-cause mortality, in different patient populations a U-shaped correlation with adverse outcomes and blood pressure is also reported [29]. Nevertheless, it is possible that endothelial dysfunction might contribute to the increased blood pressure observed in ischemic stroke patients.

Ischemic stroke is accompanied by profound changes in humoral and cellular immune responses [14,31,32,33]. Proinflammatory cytokines such as IFN-gamma, IL-6, IL-10, and TGF-beta, which are increased due to ischemic stroke, might link the brain ischemia to endothelial dysfunction [31,32,34]. Of note, sphingosine-1-phosphate (S1P) has been reported in the circulation and the ischemic brain in the 24 h following an ischemic stroke. Since S1P is established as a mediator of vascular damage, it is possible that it might induce changes in peripheral vascular responses following an ischemic stroke [33]. 

Our results implicate a humoral factor that is induced by ischemic stroke with a potential to induce endothelial dysfunction in peripheral vessels. In support of our findings, in previous studies, serum collected from ischemic stroke patients within the first 24 h of the onset of ischemic stroke was shown to induce an impairment of endothelial-dependent vascular responses in cerebral and mesenteric arteries [35]. In addition, the impairment of endothelium-dependent vascular responses was also reported with serum isolated from stroke patients with atherosclerosis 7 days to a year after the onset of the ischemic event [36]. Although our study and previous studies implicate cerebral ischemia as a triggering factor for subsequent endothelial dysfunction, whether the brain is the source of the humoral factors that trigger endothelial dysfunction remains unexplored. In one study, atherosclerotic plaque-associated proinflammatory factors such as IL-1β and the subsequent activation of cyclooxygenase are implicated [36] as triggering factors for remote endothelial dysfunction in stroke patients, although in our study, in the absence of atherosclerosis, we still observed endothelial dysfunction. Cerebral ischemia-induced endothelial dysfunction has also been associated with proinflammatory milieu and increased oxidative stress [35], increased IL-1β and cyclooxygenase products [36], and alterations in circulating white blood cells [37]. Ischemic stroke triggers the activation of neutrophils, which produce reactive oxygen species, release proteases, secrete pro-inflammatory cytokines and chemokines, and form NETs [14]. These mediators contribute to vascular damage and increase the risk of hypertension [38]. While post-stroke endothelial impairment is associated with circulating white blood cell activation [37], neutrophil-depleted serum can also induce endothelial dysfunction [5].

Cerebral ischemia accompanies an increase in the expression of endothelial adhesion molecules in the vasculature [39]. Although prior studies have mostly been limited to modifications of cerebral adhesion molecules, it is possible that cerebral ischemia may induce endothelial cell adhesion molecules in distant vasculature. For example, the increase in the adhesion molecule VCAM-1 was associated with lower flow-mediated vasodilation in stroke patients [18]. The increased expression of endothelial adhesion molecules such as β2-integrins (CD11/CD18), ICAM-1, and *p*-selectin might play a role in the recruitment of leukocytes as well as platelets in the peripheral vessels following ischemic stroke [18] and potentially might lead to cell-mediated dysfunction of endothelial cells. 

Our study discovered a diminished vasoconstrictor response to the alpha-1 agonist phenylephrine in aortas isolated from the stroke animals, compared to the sham group animals. This represents a novel finding. Alpha-1 receptors are prominent in mice aorta [40] and the activation of these receptors by selective alpha-1 agonist phenylephrine induces vascular smooth muscle contraction and vasoconstriction [41]. Our study shows that a 1.5-fold increase in phenylephrine concentration is needed to achieve the same level of vasoconstriction in aortas isolated from stroke animals in comparison to sham group aortas. These results indicate a blunted vasoconstrictor response to the alpha-1 adrenergic agonist following ischemic stroke. A reduction of in response to alpha-1 receptor agonist-induced vasoconstriction might be due to decreased alpha-1 receptor activity in response to proinflammatory cytokines. In support of this contention, phenylephrine-mediated vasoconstrictor responses were reported to be blunted with the treatment of isolated aortas with IL-6 [42].

Sympathetic nervous system induces vasoconstriction via activating alpha-1 receptors by releasing norepinephrine and epinephrine. Minute-to-minute regulation of blood pressure is achieved by the baroreceptor reflex mechanism. In the case of low blood pressure, an increase in sympathetic outflow and the subsequent activation of alpha-1 receptors on the vascular smooth muscle results in vasoconstriction, which increases total peripheral resistance and reverts the blood pressure back to normal values. Our results show that vascular smooth muscle vasoconstriction in response to alpha-1 adrenergic receptor activation is blunted following ischemic stroke. An impaired vasoconstrictor response to alpha-1 agonists after ischemic stroke might be one of the mechanisms that would impair blood pressure regulation in ischemic stroke patients. We observed an approximate 10% reduction in the vasoconstrictor response to the same concentration of phenylephrine in aortas isolated from the stroke animals. This 10% reduction in vessel size could result in around a 30% decrease in blood pressure, provided that flow is constant to the vascular beds. The implications of this blood pressure reduction following ischemic stroke may include impaired cerebral perfusion and potential hemodynamic instability. This instability poses a significant risk to stroke patients who are already vulnerable to immobility, secondary infections, and increased deep vein thrombosis. In ischemic stroke patients, hypotension is accompanied by ischemic stroke and is an independent prognostic factor for poor outcomes [29,43]. Our results suggest that impaired alpha-1 adrenergic receptor-mediated vasoconstriction might be one of the factors that lead to hypotension following ischemic stroke. Although larger patient groups are needed for conclusive results [44], the pharmacological elevation of blood pressure in the acute phases of ischemic stroke using phenylephrine appears to be associated with a better prognosis in patients [45,46].

In our study, the vasodilatory responses to nitric oxide-releasing SNP [47] were identical in vessels isolated from both the sham group and stroke animals. This finding indicates that nitric oxide-dependent vasorelaxation is preserved after ischemic stroke in first 24 h after the onset of cerebral ischemia. NO activates soluble guanylyl cyclase (sGC) and increases cGMP levels, which leads to vascular smooth muscle relaxation [41]. The preservation of vasodilatory responses to NO suggests that downstream events to NO production such as guanylyl cyclase activity, cGMP production, and cGMP-coupled vascular smooth muscle relaxation dynamics are conserved after ischemic stroke.

The peripheral vasomotor responses following a stroke may also be heavily influenced by the interference with normal angiotensin-converting enzyme-2 (ACE-2) activity. ACE-2 plays a crucial role in regulating the balance between Angiotensin-1–7 and Angiotensin-2, with Angiotensin-1–7 generally promoting vasodilation and Angiotensin-2 supporting vasoconstriction. After a stroke, the impairment of ACE-2 function could skew this balance towards a vasoconstrictor-dominant environment. This shift results in heightened vascular resistance and reduced vasodilation capacity, contributing to the compromised vasomotor responses observed in peripheral arteries post-stroke.

Additionally, the aberrant ratio of Angiotensin-1 to Angiotensin-2 not only favors vasoconstriction but also promotes hypercoagulation and inflammation, further exacerbating vascular dysfunction [42]. The increase in Angiotensin-2 can lead to the activation of pro-inflammatory pathways and the stimulation of pro-thrombotic mechanisms, thereby heightening the risk of clot formation and inflammatory damage within the vasculature. These combined effects of impaired ACE-2 function create a hostile vascular environment that may contribute to the increased blood pressure variability and overall vascular instability seen in patients after an ischemic stroke. Additionally, disturbances in the receptor for Ang-1–7, the Mas receptor, may also be a suppressed target after stroke, which provokes these types of responses when dysregulated, possibly in response to inflammatory cytokines, which may govern the expression of ACE-2, Ang-1–7, the Mas receptor, or other components of this signaling module [42,43,44,45,46].

Our study is limited by the use of thoracic aorta as the target of vascular alterations following ischemic stroke. Given that the aorta functions as a transportation vessel rather than a resistance artery, it is possible that the vascular responses we elicited may exhibit a different profile compared to resistance arteries. It is documented that arteries with vascular resistance elements such as mesenteric, splenic, hepatic, and distal omental arteries have a higher density of alpha-adrenergic receptors than the aorta [48]. It is plausible that these arteries would exhibit pronounced responses to adrenergic receptor dysfunction following ischemic stroke. Similar studies in resistance arteries remain warranted. In this study, we have not included potential humoral and neuronal signaling mechanisms that could lead to the peripheral vascular changes associated with ischemic stroke. The vascular dysfunction associated with ischemic stroke might be related to an alteration in neuronal input to the peripheral tissues, such as dysfunction of autonomic nervous system. Furthermore, since ischemic stroke is associated with a profound modification of immune system responses [32], it is possible that endothelium or vascular smooth muscle dysfunction could be a by-product of the ongoing inflammatory changes during a stroke. In addition, the possible changes in cardiac function [49] that might lead to the blood pressure variability associated with ischemic stroke have not been addressed in this study. 

**Figure 8:**
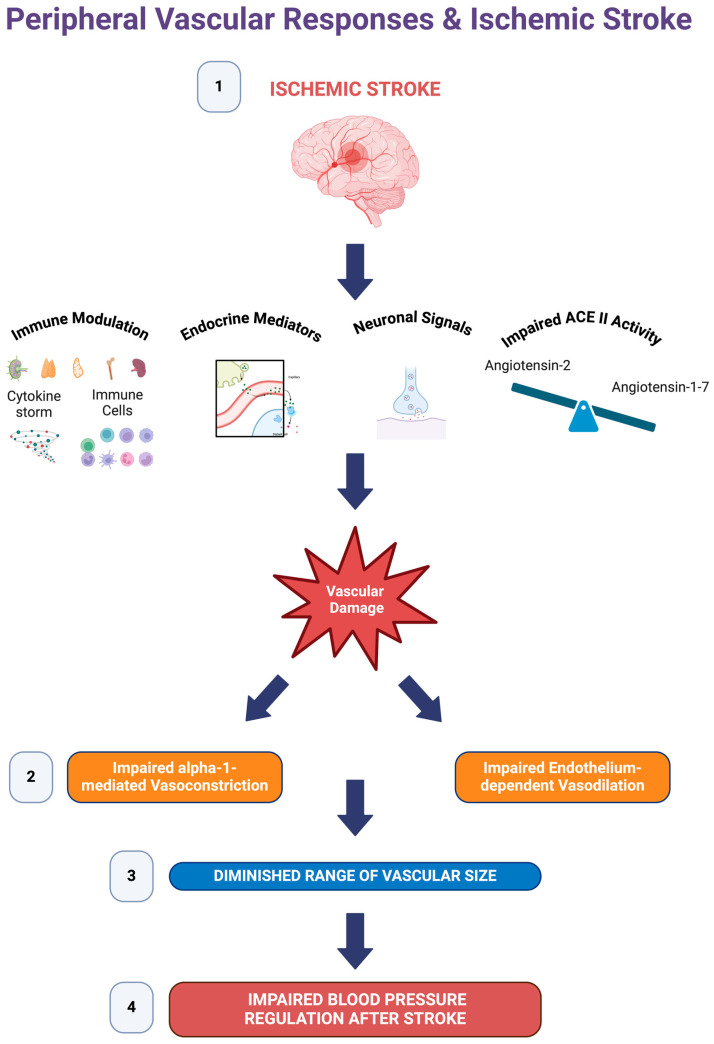
A notional summary of vascular alterations following ischemic stroke. (**1**) Ischemic stroke causes immunomodulation, the release of endocrine factors, the modulation of neuronal signals, and an imbalance of Angiotensin-1–7 and Angiotensin-2 that potentially damages peripheral vessels. (**2**) Ischemic stroke impairs alpha-1-mediated vasoconstriction and endothelium-dependent vasodilation. (**3**) The impairment of vasoconstriction and vasodilation diminishes the range of vascular sizes and, as a result, impairs the regulation of vascular resistance in response to blood pressure changes. (**4**) Due to the reduced range of vascular reactivity, blood pressure regulation is impaired following ischemic stroke. Created with BioRender.com.

Our results suggest that both vasodilatory and vasoconstrictor responses are impaired following ischemic stroke. Blood pressure variability following ischemic stroke is associated with poor clinical outcomes and higher mortality [7,10,11]. Our findings might explain the increased blood pressure variability following ischemic stroke. Local vascular changes in arteries would explain the blood pressure changes and increased blood pressure variability following an ischemic stroke. A reduced capacity in alpha-1 receptor-mediated vasoconstriction would partially impair the ability of the vascular system to increase the peripheral resistance and reset of the blood pressure to the normal physiological range when needed, whereas a reduced capacity of vasodilation would impair the reduction of vascular resistance in response to the increased blood pressure. Since both the vasoconstrictor and vasorelaxant properties of the arteries are impaired, blood pressure dysregulation following ischemic stroke might be partially explained by the impairment of local vascular responses in stroke patients (Figure 8). For reduced morbidity, it is vital to keep the blood pressure controlled, not only following ischemic stroke but also after endovascular therapies for stroke treatment [50]. Our findings might shed light on the mechanisms of blood pressure control associated with ischemic stroke. 

## Data Availability

Dataset available on request from the authors.
